# Enhancement of Corn Flour with Carob Bean for Innovative Gluten-Free Extruded Products

**DOI:** 10.3390/foods13213352

**Published:** 2024-10-22

**Authors:** Marta Igual, Rosa M. Cámara, Francesca Fortuna, Patricia García-Herrera, Mercedes M. Pedrosa, Purificación García-Segovia, Javier Martínez-Monzó, Montaña Cámara

**Affiliations:** 1 i-Food Group, Instituto Universitario de Ingeniería de Alimentos-Food UPV, Universitat Politècnica de València, Camino de Vera s/n, 46022 Valencia, Spain; marigra@upvnet.upv.es (M.I.); pugarse@tal.upv.es (P.G.-S.); xmartine@tal.upv.es (J.M.-M.); 2 Nutrition and Food Science Department, Pharmacy Faculty, Universidad Complutense de Madrid, Pza. Ramón y Cajal s/n, 28040 Madrid, Spain; rosacama@ucm.es (R.M.C.); francesca.fortuna1@studenti.unipr.it (F.F.); patrigar@ucm.es (P.G.-H.); 3 Dipartimento di Scienze degli Alimenti e del Farmaco, University of Parma, 43126 Parma, Italy; 4 Departamento Tecnología de Alimentos, Instituto Nacional de Investigación y Tecnología Agraria y Alimentaria INIA-CSIC, Ctra. de La Coruña km 7.5, 28040 Madrid, Spain; mmartin@inia.csic.es

**Keywords:** plant foods, bioactive compounds, functional foods, extrusion

## Abstract

The aim of this work is to study new, extruded products based on corn flour enriched with carob bean and the evaluation of its functional quality to develop novel gluten-free food products. Five samples based on corn flour with added carob bean flour (5 to 12.5%) were formulated. Extrusion was performed using a single-screw laboratory extruder at pilot plant scale. Extrusion parameters such as color and carbohydrate content (fiber, sucrose, and starch) were evaluated. Carob bean addition led to an increase in starch, soluble fiber, and insoluble fiber. Texture parameters related to hardness (crunchiness) were significantly reduced with the addition of CB (*p* < 0.05), detectable from a 5% addition of CB and not significant with more CB content. Samples became browner with the addition of CB; however, when the concentrations of CB are high (>5%) no major differences in color were observed. The extrusion process reduced the content of soluble and insoluble fiber, and sucrose in all formulated samples. Extruded samples with 5–7.5% CB seem to be the best formulation in terms of fiber content, color, and texture parameters. These innovative gluten-free foods could be considered as a source of fiber, and a healthier alternative to some commercially available snacks.

## 1. Introduction

Celiac disease (CD) and non-celiac gluten sensitivity (NCGS) are immune-mediated systemic disorders elicited by gluten and related prolamins, among which deamidated gliadins are the most immunogenic proteins. Immune mediated mechanisms triggered by gluten evoke intestinal mucosal damage resulting in villous atrophy in patients with CD [1]. Cereal gluten intake is the most common cause of gastrointestinal malabsorption in childhood, affecting children and adults for a lifetime. Epidemiological analysis on populations living in western countries reported that 1% of the European population suffers from CD [2], while the prevalence of NCGS is still far from being established, ranging from 1% to 6% [1]. Typically, it is characterized by chronic diarrhea, growth retardation, poor appetite, abdominal distension, and muscle weakness. The removal of gluten from the lifetime diet is the main therapy [3].

With independence from the health issues, the technological quality of baked cereal-based products, in terms of viscosity, elasticity, and cohesion, is strictly dependent on gluten. Indeed, its capacity to create protein aggregates and thus to confer structure and texture to the dough is a relevant feature for the quality of baked products. Producing gluten-free baked goods with preserved palatability and a good nutritional profile is a current challenge for the food industry [4,5]. The absence of gluten makes technological processes that affect the structure and consistency of the final product as well as the taste difficult. Gluten-free bakery products often have an elevated glycemic index and lower nutritional value compared with gluten-containing products. To solve this problem, the food industry takes into account the use of a combination of several ingredients, looking for different types of starch or gluten replacers, that provide not only better nutritional properties but also good taste, appearance, and shelf life [6].

The first approach in the processing of these products is the use of gluten-free flours containing various protein sources as alternatives to wheat flour. Ingredients allowed in a gluten-free diet include rice, corn, soy, pea, millet, potato, sorghum, amaranth, quinoa, and buckwheat flours and by-products from those raw materials [7]. In addition to technological limitations, the lack of compliance with taste, insufficiency of gluten-free products in the market, and their high cost are still problems to solve. The use of corn products in gluten-free foods is common as the properties of gluten-free foods obtained from corn could have similar quality attributes compared to the foods made from wheat flour for the general population, with the exception of color. Although, for some individuals the very strong color and taste characteristics of corn could be a disadvantage [8,9].

Carob (*Ceratonia siliqua* L.) is an evergreen tree that belongs to the Leguminosae or Fabaceae family (subfamily Caesalpinioideae) native to the Mediterranean region. It has been widely exploited since antiquity due to its edible fruits (commonly referred to as pods or merely carobs). However, currently, its fruits are used for animal feed, and new alternatives are being sought to promote its human consumption. Lobanov et al. [10] studied the impact of incorporating vegetal ingredients in the innovative production of flour-based functional foods. In particular, the enhancement of carob bean resulted in an increased content of proteins, fat, and fibers, and a decreased carbohydrate content. Thus, carob bean can be an excellent raw material to produce gluten-free flours enriched with vitamins, minerals, and proteins [11].

Over the recent decades, our diet within westernized societies has changed radically from that of our ancestors, with implications for our co-evolved gut microbiota. Changes include a reduction in the intake of fiber-replete plant-based foods. Lack of fiber in the Western diet poses not only nutritional but also health risks, which is why different strategies are being sought to increase the fiber content of food. In order to improve fiber intake in the population, formulating snack-type products containing dietary fiber could be of interest, as it will increase food diversity as well as confer several health benefits [12]. The lack of fiber in gluten-free baked and snack products has been subjected to improvement in the formulation of these products, with the addition of corn, sugar beet fiber, flaxseed fiber, pseudocereals or hydrocolloids, among other sources of fiber. Carob bean flour has been recently attracted great interest for the formulation of gluten-free products, due to its nutritional value not only as a fiber source [13].

Carob bean presents insoluble and non-fermentable fiber (cellulose, hemicellulose and lignin). This fiber improves gastrointestinal functionality, increases the feeling of satiety, and presents antidiabetic and anti-obesity effects. Also, it contains small amounts of soluble dietary fiber as pectins. In this case, fiber acts as a prebiotic due to colon fermentation [14]. Thus, carob bean as a food ingredient could be an innovative solution not only to increase the fiber content of formulations, but also allows for the obtaining of suitable food products for people with gluten-related issues. In addition, complementary formulations based on cereals (as corn) and pulses (as carob bean) are of interest for the final nutrient composition (mainly the essential amino acids) as well as for improving pulse consumption in populations using innovative food products which could replace energy-dense snacks with more nutritious ones for the young and adult populations [15].

The aim of this work is to study new extruded products based on corn flour enriched with carob bean, and the evaluation of the functional quality of extruded products obtained based on their fiber contents, to develop novel gluten-free food products.

## 2. Materials and Methods

### 2.1. Sample Materials and Extrusion Process

In the present study, five flour formulations based on corn flour with added carob (from 5 to 12.5%) were performed (Table 1). Maicerías Españolas S.L. (Valencia, Spain) supplied corn grits (CM) and Alpi Investment OOD (Sofia, Bulgaria) supplied carob bean (CB) powder. Corn flour was mixed manually using a whisk, with increasing CB powder percentages of 5, 7.5, 10, and 12.5% to obtain the enriched flour formulations. The water content of the mixtures ranged between 11 and 12%.

Samples were processed at the i-Food Group, IIAD, Universitat Politécnica de Valencia, Camino de Vera s/n, 46,022 Valencia, Spain.

Extrusion was performed using a single-screw laboratory extruder (Kompaktextruder KE 19/25; Brabender, Duisburg, Germany) with a barrel diameter of 19 mm and a length/diameter ratio of 25:1. Extrudates were cooled at ambient temperature (23 °C), then grounded and sealed in plastic bags for further analysis. The names and codes of the unprocessed and extruded samples are given in Table 1.

**Figure 1:**
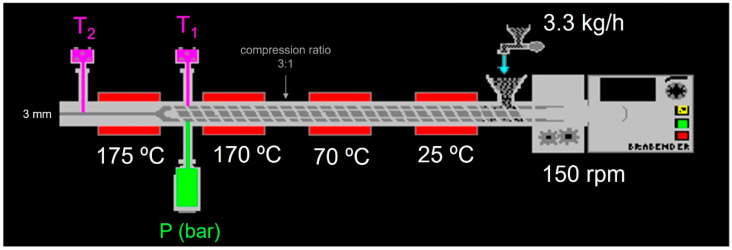
Scheme of conditions used in the extruder.

Figure 1 shows the conditions during extrusion. Screw speed, motor torque, melt pressure (P), and barrel temperatures (T_1_ and T_2_) were monitored using Extruder Winext software (Brabender).

### 2.2. Analytical Determinations

#### 2.2.1. Extrusion Parameters

One of the most interesting properties in extruded products is expansion. This can be measured by different parameters, among which the surface expansion index of the die (SEI) and bulk density stand out (ρ_b_). SEI was calculated as the quotient between the square of the measured extrudates diameters and the square of the die diameter [16]. A total of 20 diameters of extruded pieces were measured for each sample with an electronic Vernier caliper (Comecta S.A., Barcelona, Spain). ρ_b_ is considered as expansion in all directions, different to SEI, which considers expansion only in the direction perpendicular to the extrudate flow [17]. For ρ_b_ determination, measurements were taken 14 times, where the diameter and height of cylinders were measured with an electronic Vernier caliper (Comecta S.A., Barcelona, Spain) and samples were weighed with a precision scale (± 0.001 g) (Mettler Toledo, Greifensee, Switzerland).

The porosity (ε), percentage of air volume related to the total volume, was calculated from the true (ρ) and bulk (ρ_b_) densities according to [18]. ρ of the extruded products was determined using a helium pycnometer in triplicate (AccPyc 1330, Micromeritics, Norcross, GA, USA).

To evaluate the hydration properties, the water absorption index (WAI) and water solubility index (WSI) were used. WSI and WAI were determined by the method of Singh et al. [19] and calculated according to Uribe-Wandurraga et al. [20]. Swelling index (SWE) also was measured using the bed volume technique. The bed volume was recorded and expressed as mm of the swollen sample per g of the dry initial sample [18].

Hygroscopicity (Hy) was determined according to Cai et al. [21]. Extruded samples were placed in a Petri dish by triplicate at 25 °C, in an airtight plastic container containing Na_2_SO_4_ saturated solution (81% relative humidity). After 7 days each sample was weighed and the hygroscopicity (Hy) was expressed as g of water gained per 100 g dry solids.

Texture properties were measured using puncture tests with a TA-XT2 Texture Analyzer (Stable Micro Systems Ltd., Godalming, UK) and software, Texture Exponent (version 6.1.12.0). A 2 mm-diameter cylinder was used, and the crosshead speed was kept at 0.6 mm/s [18]. From the force–time curve, the area under the curve plot which represented work performed, a time of displacement of the puncturing device was obtained from extrudates (8 times). The force drop of each peak was also obtained and it represented the local resistance of cell walls, and the number of peaks (No) were also recorded [22]. These parameters were used to calculate the average puncturing force (Fp), average specific force of structural ruptures (Fs), spatial frequency of structural ruptures (Nsr), and crispness work (Wc) according to Igual et al. [23].

#### 2.2.2. Color Attributes

Color was determined in powdered samples. Flours and extrudates were grounded and placed in a cup specially designed for this purpose. Color was measured using Minolta spectrophotometer CM-3600d (Japan). CIE*L*a*b* color coordinates were determined considering standard light source D65 and standard observer 10° in mixtures and extrudates (8 times). Hue (h*), and chroma (C*) color attributes were calculated from CIE*L*a*b* color coordinates. The total color differences in mixtures or extrudates with CB (ΔE_1_) were calculated for the control sample. To evaluate the color changes in the mixtures because of extrusion, total color difference (ΔE_2_) was calculated between each mixture and extrudate at same CB % addition [18].

#### 2.2.3. Water Content and Water Loss

Water content (x_w_) (g/100 g) was determined using vacuum oven drying at 105 °C until constant weight was achieved [21] for mixtures with and without CB % and extruded samples by triplicate. Samples were analyzed in triplicate. Water loss (W_L_) because of the extrusion process was calculated.

#### 2.2.4. Fiber Content

The fiber content in the samples analyzed was determined by triplicate, by the AOAC enzymatic–gravimetric method (Ref. 993.19 and 991.42) [24], quantifying insoluble, soluble, and total fiber. The basis of this method is the enzymatic digestion of samples with α-amylase, protease, and amyloglucosidase (Sigma-Aldrich, St. Louis, MO, USA) to remove both protein and starch present. Liquid obtained was filtered with a Gooch Pyrex filter plate, and then the residue collected was dried to 100 °C and weighed with the aim of obtaining the insoluble fiber. A total of 400 mL of ethanol (96% *v*/*v*) was added to the liquids for which insoluble fiber was filtered, and they were stored to the next day to precipitate the soluble fiber. Then, they were filtered with the same conditions as for insoluble fiber. In both cases, ashes and proteins were analyzed and residues were corrected by ash and protein content.

#### 2.2.5. Soluble Sugars

Extraction of sugars was performed with a mixture of ethanol/water in a proportion of 50%. Free sugars extracted were analyzed with a HPLC system coupled to a refractive index detector (Beckman System Gold Instrument, Los Angeles, CA, USA) following the method described by Pedrosa et al. [25]. The amount of 20 µL of each sample was injected in a spherisorb-5-NH2 column (250 × 4.6 mm i.d., Waters, Milford, MA, USA) equilibrated with acetonitrile/water (60:40, *v*/*v*) at a flow rate of 1 mL/min. Calibration curves from external standards (Sigma, St. Louis, MO, USA) were used for identification and quantification.

Unprocessed and extruded samples of raw corn and carob bean, and formulated samples, were analyzed by triplicate.

#### 2.2.6. Total Starch Content

After free sugar extraction, the residue was analyzed for total starch content using a Megazyme kit (Wicklow, Ireland). This kit contains an improved α-amylase that allows for starch hydrolysis with amylase incubations at pH 3.8. Glucose was determined by spectrophotometry at 510 nm in samples according to Arribas et al. [26] based on AOAC method 996.11 and 76.13.01. Unprocessed and extruded samples without carob bean formulation and with the with the higher amount of CB formulated (12.5%) were analyzed in triplicate.

### 2.3. Statistical Analysis

Analysis of variance (ANOVA), with a confidence level of 95% (*p* < 0.05), was applied using Statgraphics Centurion XVII, version 17.2.04, to evaluate the differences among mixtures or extruded samples and to evaluate the extrusion process. Equations fitted significantly (*p* < 0.05) for WAI, WSI, SWE and Hy against CB concentration were also obtained using Statgraphics Centurion XVII software. The method used to discriminate among means was Fisher’s Least Significant Difference procedure. A correlation analysis was performed with the extrusion parameters and the textural properties of the extrudates produced, with a significance level of 95% (Statgraphics Centurion XVII, Statgraphics Technologies, Inc., The Plains, VA, USA).

## 3. Results

### 3.1. Extrusion Process Parameters

The extrusion process was monitored, and the parameters were obtained for each sample. P, T_1_, and T_2_ are listed in Table 2 and were illustrated in Figure 1. Results obtained are in the range observed in other corn-based work carried out with the same extruder [18,23,27]. Specific mechanical energy (SME) can be defined as the energy required for the production of 1 g of extrudate [28]. SME was calculated according Logi et al. [29]. Water loss for extrusion (W_L_) is also shown in Table 2. The addition of CB significantly (*p* < 0.05) increased T_2_ and decreased SME, while for other parameters, such as T_1_ or W_L_, slight differences with F0 were observed but without a defined trend.

Other studies have shown that feed composition significantly affects SME. Specifically, they indicate that higher protein contents in mixtures caused lower SME [30]. This is probably the reason why the sample F0, without CB and with a higher corn content than the rest, and therefore with lower protein content, showed significantly higher SME values.

The parameters that characterize the snacks obtained by direct expansion are those related to their size increase at the nozzle exit. Expansion occurs due to the pressure drop caused by the passage of the melt mass from elevated pressure to atmospheric pressure as it leaves the sealed matrix. Table 3 shows the SEI, ρ_b_, and ε values for the extrudates obtained.

Highly expanded extruded materials exhibit a porous structure. This structure is produced by processing, which causes changes in the cell structure, pores and internal voids of the matrix [31]. To evaluate the volume of occluded air in extrudates, ε values are studied. SEI was significantly (*p* < 0.05) affected by the addition of CB. However, ρb and ε showed no significant (*p* > 0.05) differences. The values obtained for these parameters were similar to those presented by other studies [18]. In general, the addition of CB did not drastically affect the expansion in comparison with other studies in which the addition of lucerne or nettle showed significant and very noticeable changes in SEI, ρ_b_, and ε [18,23]. In these studies, an effect of the % of lucerne and nettle was also observed in the extrudates, respectively; however, in the present study, it was not evident for these parameters. Probably because the fiber content of CB is lower than that of nettle or alfalfa.

### 3.2. Water Absorption Index (WAI) and Water Solubility Index (WSI) of Formulated and Extruded Samples

**Figure 2:**
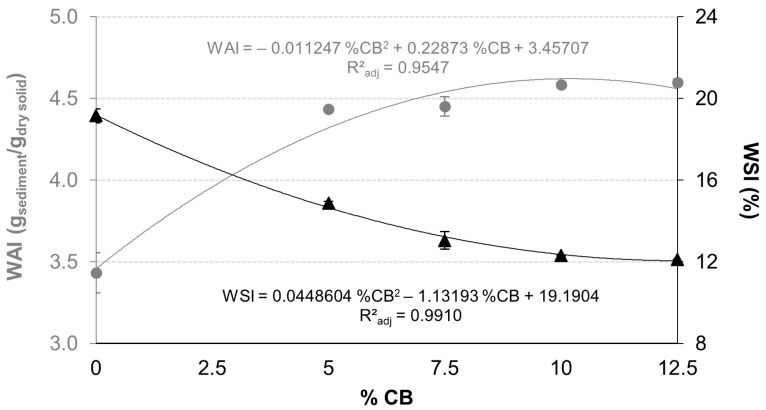
Mean values and standard deviation of water absorption index (WAI) and water solubility index (WSI) of extrudate according to carob bean (CB) %. Figure includes the significantly (*p* < 0.05) fitted and R^2^-adjusted equation.

**Figure 3:**
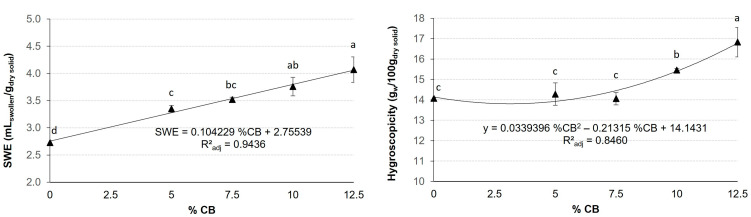
Mean values and standard deviation of swelling index (SWE) and hygroscopicity of extrudate according to carob bean (CB) %. Figure includes the equation fitted significantly (*p* < 0.05) and R^2^ adjusted. Letters indicate homogeneous groups established by the ANOVA (*p* < 0.05) for each parameter analyzed.

Mean values and standard deviations of the WAI, WSI, SWE, and Hy of extrudates are presented in Figure 2 and Figure 3. These values were fitted to a polynomial equation with a significance level of 95%. These equations are close to the experimental data, as can be seen from the adjusted R^2^. WAI, WSI and Hy are represented by the equation with a CB% simple effect and CB% quadratic effect in the studied range (0–12.5%). SWE is represented only by the equation with a CB% simple effect; however, there is no significant CB% quadratic by SWE.

Figure 2 shows that the addition of increasing concentrations of CB increased WAI and, contrarily, decreased WSI. WAI and WSI report physico-chemical changes in biopolymers due to the extrusion processing [32]. WAI indicates the amount of water immobilized by the extrudate [33], while WSI indicates the amount of small molecules solubilized in water causing molecular damage in the process [34]. Therefore, CB in extruded mixtures reduces the risk of possible molecular damage by water-solubilized molecules. This indicates that CB causes less solubilization of matrix components during extrusion, mainly by showing less starch degradation towards the formation of soluble fragments.

SWE (Figure 3) gradually increases with increasing CB% in the mixtures. The addition of CB significantly (*p* < 0.05) increased the SWE value. However, when comparing the samples containing CB, the effect of the % is significantly (*p* < 0.05) seen when there is an increase of 5% or higher. That is, significant (*p* < 0.05) differences are observed between the extrudates of 5 and 10% or higher concentration, and between 7.5 and 12.5%.

Hy is also shown in Figure 3. Hy did not show significant (*p* > 0.05) changes in formulations containing up to 7.5% CB. Above this % of CB, Hy increased significantly (*p* < 0.05). Other nettle-enriched snacks also showed higher Hy when nettle concentrations were higher [30]. This effect may be due to the fiber content of the samples.

Table 4 shows the textural properties of the extrudates obtained. Texture is one of the most outstanding properties in a snack obtained by extrusion expansion [35]. The parameters shown in Table 4 for F0, without CB, are similar to those shown in other studies for the control of corn snacks [18]. The addition of CB significantly (*p* < 0.05) reduced the texture parameters related to hardness (Wc, Fs and Fp) and increased the values of texture parameters related to crunchiness (Nsr and N0). In the Wc values, the significant (*p* < 0.05) effect of CB addition is detectable from 5% (F1), then in the range of 7.5–12.5 (F2–F4) no significant (*p* > 0.05) differences were observed among these samples. In the case of Fs and Fp, the significant (*p* < 0.05) differences are from 7.5% of CB (F2).

The parameters determined in the extruded products have been related by Pearson correlations to explore their relationship. Table 5 shows Pearson’s coefficients obtained for the correlations between the parameters studied in the extruded products and also the CB %.

The CB concentration in the formulation of the extruded snacks was significantly correlated (*p* < 0.05) with several of the parameters determined in the products. Increasing CB% in the snacks increased WAI and SWE but decreased x_w_, WSI, Wc, Fs, Fp, and SEI. In this sense, the incorporation of higher concentrations of CB produces crispier snacks that are able to absorb water, but are less hard, dry and expanded. These trends are caused by the composition of CB, in particular by its insoluble fiber content. Textural parameters are closely related to the parameters describing the behavior of the snack upon hydration. Thus, the following relationship is established: as the water absorption or swelling capacity of the snacks increases, the hardness of the snacks decreases, and the crispness work of the snacks is reduced. SME dissipated during shearing is transformed into thermal energy, increasing the temperature of the material and modifying the physical and chemical properties of the extruded food [36]. With the decrease in SME input, the textural properties of products such as hardness and chewiness also decreased significantly [37], as can be observed in the trends of samples in Table 1 and Table 4.

As can be seen in Table 5, SME is significantly (*p* < 0.05) and positively correlated with WSI, SEI, ε, and Wc, and negatively correlated with WAI, SWE, Nsr, and N0. A higher SME value is related to a decrease in the crispness of the snacks obtained, despite obtaining more porous and expanded snacks. Table 5 shows that there is a high significant (*p* < 0.05) correlation between snack expansion and moisture in the same sense as shown by Igual et al. [23], and also with the indices related to snack water uptake (WAI, WSI, and SWE) as shown in other works [18].

### 3.3. Color Determination

**Figure 4:**
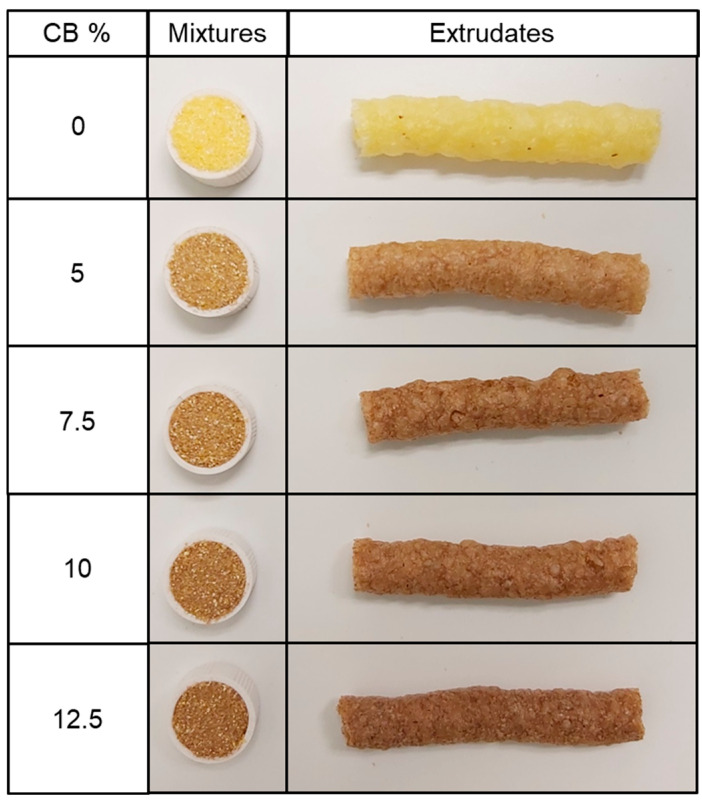
Appearance of studied unprocessed mixtures and extrudates of corn flour with different concentrations (0, 5, 7.5, 10, 12.5, and 15%) of carob bean (CB).

The incorporation of carob bean in the corn base formulation to obtain extrudates considerably affected the color of the mixtures and extrudates, as can be seen in Figure 4.

Instrumentally, the mean values and deviations of the color coordinates and the color differences can be seen in Table 6. In both mixtures and extrudates, the addition of CB significantly (*p* < 0.05) decreased the L* values due to the characteristic dark color of CB flour. This effect is more marked in the mixtures, as the extrusion process generates a significant (*p* < 0.05) L* reduction in all cases, as has been showed by other works [18,23]. The a* coordinate showed small changes between the mixture samples (F0–F4); however, a* increased significantly (*p* < 0.05) with increasing CB in the extrudates (F0E–F4E). b* decreased significantly (*p* < 0.05) in the mixtures and in the extrudates with increasing CB content. C and h also decreased significantly (*p* < 0.05) with CB incorporation. The samples become browner with the addition of CB; however, when the concentrations of CB are high (F2–F4), no major differences are observed for the coordinates studied. The differences in color due to the incorporation of CB (ΔE_1_) in the mixtures and in the extrudate showed high values; however, no statistical (*p* > 0.05) differences in ΔE_1_ were observed in samples F2, F3, and F4. The differences in color due to the effect of extrusion (ΔE_2_) were markedly and significantly (*p* < 0.05) higher in the sample without CB (F0) compared to the rest. The other samples were ordered from lowest to highest ΔE_2_ according to their CB %.

### 3.4. Carbohydrate Fraction: Free Sugars, Fiber, and Starch Content

**Figure 5:**
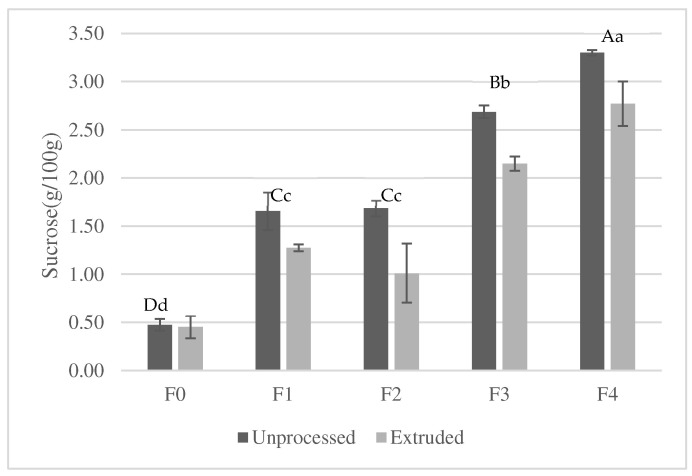
Sucrose content in unprocessed and extruded samples of formulations (g/100 g, dry weight) (mean ± SD). The same capital letter within the black column indicates homogeneous groups, as established by ANOVA (*p* < 0.05), comparing CB% (0, 2.5, 5, 7.5, 10, 12.5, and 15) in unprocessed mixtures. For each CB % (0, 2.5, 5, 7.5, 10, 12.5, and 15), the same small letter in gray bars indicates homogeneous groups as established by ANOVA (*p* < 0.05), comparing mixtures and extrudates.

Free sugars of unprocessed and extruded samples were analyzed, with sucrose being the main free sugar quantified, as can be seen in Figure 5. The sucrose content of unprocessed corn flour is 0.47 g/100 g (F0), while carob bean flour has a sucrose content of 58.73 g/100 g. The addition of carob bean flour to the formulations results in an increase in the sucrose content up to 3.30 g/100 g in the F4 sample, corresponding to 87.5% corn flour and 12.5% carob bean. Extruded samples formulated with carob bean flour showed less sucrose content than unprocessed samples due to the Maillard reaction that took place in the extrusion process at high temperatures, with a consequent reduction in the content of simple sugar [38]. The loss of sucrose content due to the extrusion process ranged between 16 and 40% in samples formulated with carob bean (F1 to F4).

Regarding Fiber content (Table 7), corn flour (F0) is characterized by a total fiber content of 8.2 g/100 g, with insoluble fiber (IDF) being the predominant fraction (90%), in comparison with soluble fiber (SDF). The carob flour was also analyzed with a result of a very high content of fiber (IDF = 34.36 ± 4.06; SDF = 10.25 ± 0.40; TDF = 42.51 ± 3.97); thus, it is interesting to consider this ingredient as a good source of fiber to be added to food to increase the low daily intake of dietary fiber. As was expected, samples enriched with Carob bean flour (F1 to F4) showed significantly higher levels of TDF than the F0 control sample, up to 75%. It can also be seen that increasing the addition of carob flour in the samples leads to a constant increase in fiber content in both extruded and raw samples.

An important increase in insoluble, soluble, and total fiber from the pure sample without carob (F0) to the sample with 5% carob addition (F1) is evident, being an increase of ˃30% for IDF and TDF and 17% for SDF. Compared to F4 (12.5% carob addition), this creates an enhancement of 47% in insoluble fiber, 40% in soluble fiber, and 44% in total fiber.

The extrusion process affects the properties and structures of fibers. Many authors found an increase in the solubility of fiber after the extrusion process [39,40,41], and thus, a decrease in its final content. However, this effect depends on the process conditions, raw material sources, and particle size of the fiber The impacts of fibers on the expansion of starch extrudates can be dependent on the types of fibers, their contents in raw ingredients, and extrusion processing conditions [42,43,44]. Extrusion may also enhance the accessibility of the insoluble fiber fraction to fermentation by gut microbiota. For example, in one study comparing unprocessed and extruded whole-grain barley, corn, oats, rice, and wheat, extrusion increased the fermentability of the nondigestible carbohydrates in all of these grains compared to the unprocessed counterpart [45]. In another study, the fermentability of the nondigestible carbohydrates more than doubled when the wheat bran was extruded versus boiled. The most effective conditions for increasing fermentation by gut bacteria were low moisture and slow screw speed [46].

As can be seen in Table 7, the addition of carob bean flour enhanced the percentage of fiber in raw and in extruded samples.

Comparing extruded versus unprocessed formulations considered in this study, the extruded products presented lower fiber content than raw products, as previously mentioned, but the addition of carob bean flour improved the fiber composition versus the non-carob bean flour sample (F0E), showing a significant increase ˃100% for IDF and TDF, and 30% for SDF in F4E. Unprocessed samples showed significant higher levels of TF and IF than the extruded ones.

The European Food Safety Authority [47] sets a dietary reference value of 25 g of fiber per day for adults and 21 g of fiber per day for young people and adolescents. Final extruded products considered in this study cover between 26 and 55% of the daily fiber requirement. And all the flours considered in this study can be considered a source of fiber (with a content of more than 3 g/100 g) according to [48], and thus are all interesting formulations for the development of functional foods.

Total starch was measured in unprocessed and processed formulations without the formulation of carob bean (F0 and F0E) and formulated with 12.5% of carob bean (F4 and F4E) in order to study the effect of the extrusion in the formulation considering the sample with higher amount of carob bean.

**Figure 6:**
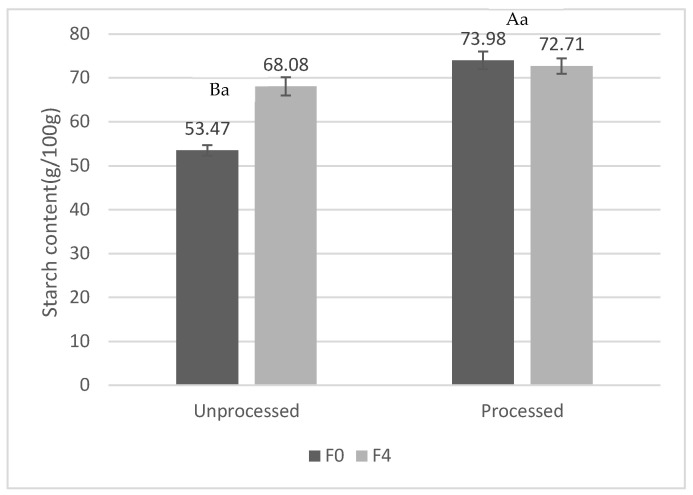
Starch content of formulation F0 (100% corn flour) and F4 (87.5% corn flour +12.5% carob bean), unprocessed and extruded (g/100 g, dry weight) (mean ± SD). The same letter in capitals indicates homogeneous groups as established by ANOVA (*p* < 0.05), between unprocessed and processed samples, while small letters refer to the effect of formulation (F0 and F4).

The incorporation of carob bean leads to an increase in total starch content of 14.61% as is shown in Figure 6. TS content is significantly more increased in non-formulated extruded samples (F0E) (20.51%) compared to in formulated ones (F4E) (4.63%). The extrusion process has a direct effect on the total amount of starch that can be gelatinized, totally or partially, or even melted or fragmented due to the extrusion conditions. The presence of some compounds such as sucrose, lipids, and other substances can cause a delay in the gelatinization process. After extrusion, partially gelatinized starch undergoes retrogradation with cooling, and lipids bind to the starch, mainly to amylose, which translates into an increase in the resistant starch amount. In addition, depending on different sources of starch, raw materials, and their distinct gelatinization/retrogradation properties, large variance in starch digestibility could be created in the extruded foods [49,50].

## 4. Conclusions

Regarding the newly formulated corn flours enriched with carob bean, the first attribute modified is color. In both mixtures and extrudates, the addition of CB leads to decreases in the L* values due to the characteristic dark color of CB flour. Texture parameters related to hardness (crunchiness), as one of the most outstanding properties in a snack, obtained by extrusion expansion, was significantly reduced with the addition of CB, detectable from a 5% CB addition, and was not significant with higher CB content. The incorporation of higher concentrations of CB produces crispier snacks that can absorb water but are less hard, dry, and expanded. CB also improves the fiber content, mainly total and insoluble fiber.

The extrusion process leads to total, soluble, and insoluble fiber decreasing in all formulated samples analyzed, when comparing to the reference flour (unprocessed). The same fact occurred with the sucrose, the main soluble sugar in the samples, which suffered losses by the Maillard reaction. Finally, the extrusion process increased the total starch amount. Extrusion may also enhance the accessibility of the insoluble fiber fraction to fermentation by gut microbiota. More studies should be realized in this sense. Extruded samples with 5–7.5% of CB seem to be the best formulation in terms of fiber content, color, and texture parameters, as nutritious gluten-free snacks. As a limitation, previously, the authors worked with higher percentages, but the limitations of the extruder meant that more CB could not be incorporated.

Extruded products considered in this study have functional qualities for developing novel gluten-free food products, as they cover between 26 and 55% of the daily fiber requirement, and thus can be considered a source of fiber. These enriched carob bean snacks meet the challenge of the aim of this work and can be considered a healthier alternative in comparison to some commercially available snacks. Sensory analysis studies will be considered in future works to establish the best formulation in terms of consumer acceptance.

## Figures and Tables

**Table 1:** Samples code and formulation composition based on corn and carob bean formulations.

Unprocessed	Extruded	Corn Flour (g/100 g)	Carob Bean Flour (g/100 g)
F0	F0E	100	0
F1	F1E	95	5
F2	F2E	92.5	7.5
F3	F3E	90	10
F4	F4E	87.5	12.5

**Table 2:** Mean values (and standard deviations) of melt pressure (P), barrel temperatures (T_1_, T_2_), specific mechanical energy (SME) and water loss (W_L_) of studied samples.

Sample	P (Pa)	T_1_ (°C)	T_2_ (°C)	SME (J/g)	W_L_ (g_w_/g_db_)
F0	84 (3) ^cd^	178.6 (0.7) ^cd^	90.8 (0.4) ^c^	1002 (5) ^a^	0.0850 (0.0009) ^b^
F1	89.3 (1.2) ^a^	177.8 (1.2) ^d^	97.0 (0.5) ^a^	771 (15) ^b^	0.077 (0.003) ^d^
F2	87 (3) ^ab^	179.3 (0.5) ^b^	96.1 (0.4) ^b^	784 (7) ^b^	0.0776 (0.0003) ^cd^
F3	86 (3) ^bc^	180.3 (0.5) ^a^	96.8 (0.4) ^a^	789 (14) ^b^	0.0815(0.0013) ^bc^
F4	82 (2) ^d^	179.1 (0.4) ^bc^	96.1 (0.04) ^b^	789 (14) ^b^	0.09168 (0.00012) ^a^

The same letter in superscript within a column indicates homogeneous groups, as established by ANOVA (*p* < 0.05).

**Table 3:** Mean values (and standard deviations) of sectional expansion index (SEI), bulk density (ρ_b_), and porosity (ε) of extruded samples (F0E–F4E).

Sample	SEI	ρ_b_ (g/cm^3^)	ε (%)
F0E	13.9 (0.6) ^a^	0.085 (0.007) ^a^	93.7 (0.2) ^a^
F1E	13.3 (0.3) ^b^	0.088 (0.003) ^a^	91.7 (0.5) ^c^
F2E	12.8 (0.3) ^c^	0.089 (0.005) ^a^	91.8 (0.7) ^c^
F3E	13.1 (0.3) ^bc^	0.0876 (0.0004) ^a^	92.28 (0.03) ^bc^
F4E	12.3 (0.6) ^d^	0.085 (0.005) ^a^	93.0 (0.5) ^ab^

The same letter in superscript within a column indicates homogeneous groups, as established by ANOVA (*p* < 0.05).

**Table 4:** Mean values (and standard deviations) of crispness work (Wc), average specific force of structural ruptures (Fs), average puncturing force (Fp), spatial frequency of structural ruptures (Nsr), and number of peaks (N0) of extrudated samples.

Sample	Wc (N*mm)	Fs (N)	Fp (N)	Nsr (mm^−1^)	N0
F0E	0.27 (0.05) ^a^	2.2 (0.4) ^a^	1.54 (0.17) ^a^	9.7 (1.3) ^b^	105 (12) ^b^
F1E	0.155 (0.014) ^b^	2.01 (0.14) ^a^	1.59 (0.15) ^a^	13.0 (0.7) ^a^	142 (12) ^a^
F2E	0.11 (0.02) ^c^	1.3 (0.2) ^b^	0.97 (0.08) ^b^	12.0 (1.5) ^a^	133 (18) ^a^
F3E	0.094 (0.009) ^c^	1.15 (0.14) ^bc^	0.86 (0.12) ^b^	12.2 (0.5) ^a^	132 (6) ^a^
F4E	0.081 (0.013) ^c^	0.95 (0.12) ^c^	0.62 (0.12) ^c^	12 (2) ^a^	122 (20) ^ab^

The same letter in superscript within column indicates homogeneous groups established by ANOVA (*p* < 0.05).

**Table 5:** Pearson correlation coefficients (* correlation is significant at 0.05) among studied parameters of extruded products and CB percentages.

	W_L_	SME	WAI	WSI	SWE	Hy	SEI	ρ_b_	ε	W_c_	N_sr_	F_s_	F_p_	N_0_	CB (%)
x_w_	−0.5806	0.7092 *	−0.7983 *	0.8375 *	−0.9316 *	−0.8073 *	0.9331 *	0.0791	0.2137	0.8270 *	−0.4902	0.7873 *	0.7714 *	−0.3091	−0.9332 *
W_L_		0.1146	0.0599	−0.1973	0.4335	0.7972 *	−0.4869	−0.3380	0.5805	−0.1860	−0.2350	−0.4089	−0.5003	−0.4376	0.4531
SME			−0.9646 *	0.8906 *	−0.7986 *	−0.3227	0.7014 *	−0.2555	0.7737 *	0.8817 *	−0.8236 *	0.6239	0.5125	−0.7695 *	−0.7674 *
WAI				−0.9600 *	0.8895 *	0.4955	−0.7879 *	0.2559	−0.6656 *	−0.9557 *	0.8256 *	−0.7412 *	−0.6369 *	0.7329 *	0.8824 *
WSI					−0.9323 *	−0.5959	0.8465 *	−0.1418	0.5075	0.9898 *	−0.6761 *	−0.8904 *	−0.8164 *	−0.5563	−0.9533 *
SWE						0.7185 *	−0.8857 *	0.1252	−0.3621	−0.9195 *	0.5211	−0.8751 *	−0.8305 *	0.3729	0.9745 *
Hy							−0.7047 *	−0.4217	0.2951	−0.5470	0.3506	−0.6161 *	−0.6487 *	0.0395	0.7851 *
SEI								−0.0156	0.2425	0.8688 *	−0.5258	0.8310 *	0.7989 *	−0.3501	−0.8898 *
ρ_b_									−0.6566 *	−0.2400	0.3307	−0.0804	0.0301	0.3636	0.0080
ε										0.5438	−0.7233 *	0.2315	0.0946	−0.7695 *	−0.2875
W_c_											−0.6984 *	0.8943 *	0.8120 *	−0.5738	−0.9340 *
N_sr_												−0.3160	−0.1719	0.9708 *	0.5336
F_s_													0.9853 *	−0.1602	−0.9110 *
F_p_														−0.0048	−0.8750 *
N_0_															0.3716

**Table 6:** Mean values (and standard deviations) of color coordinates (L*, a*, b*, C, and h) and total color differences (ΔE) of unprocessed (F0–F4) and extruded samples (F0E–F4E), with 0–12.5% CB.

	Unprocessed				
Parameter	F0	F1	F2	F3	F4
L*	80.5 (0.5) ^aA^	60.9 (0.5) ^bA^	57.83 (0.12) ^cA^	56.01 (1.02) ^dA^	54.83 (1.02) ^eA^
a*	8.3 (0.4) ^bA^	8.3 (0.4) ^bA^	8.67 (0.12) ^abA^	8.4 (0.2) ^bA^	8.9 (0.2) ^aA^
b*	41.2 (0.5) ^aA^	24.8 (0.9) ^bA^	23.5 (0.5) ^cA^	19.7 (0.2) ^dA^	19.7 (0.4) ^dA^
C	42.0 (0.5) ^aA^	26.14 (1.02) ^bA^	25.0 (0.5) ^cA^	21.4 (0.2) ^dA^	21.6 (0.3) ^dA^
h	78.5 (0.5) ^aB^	71.6 (0.3) ^bB^	69.72 (0.14) ^cA^	66.9 (0.4) ^dA^	65.8 (0.9) ^eA^
ΔE_1_	-	25.6 (0.8) ^cA^	28.9 (0.2) ^bA^	32.7 (0.6) ^aB^	33.57 (1.02) ^aA^
	Extruded				
	F0E	F1E	F2E	F3E	F4E
L*	51 (4) ^aB^	51.4 (0.5) ^aB^	43.0 (0.8) ^bB^	42 (2) ^bB^	37 (2) ^cB^
a*	1.2 (0.3) ^dB^	3.9 (0.2) ^cB^	5.5 (0.5) ^bB^	6.3 (0.6) ^aB^	5.5 (0.6) ^bB^
b*	16.7 (1.6) ^aB^	13.2 (0.5) ^bB^	8.8 (0.2) ^cB^	9.5 (1.8) ^cB^	5.93 (1.12) ^dB^
C	16.8 (1.6) ^aB^	13.8 (0.5) ^bB^	10.4 (0.4) ^cB^	11.4 (1.8) ^cB^	8.1 (1.2) ^dB^
h	85.80 (1.09) ^aA^	73.55 (0.13) ^bA^	57.8 (1.8) ^cB^	56 (3) ^cB^	47 (3) ^dB^
ΔE_1_	-	4.5 (0.4) ^cB^	12.0 (0.6) ^bB^	12 (2) ^bA^	18 (2) ^aB^
ΔE_2_	39.2 (0.2) ^a^	15.7 (0.9) ^e^	21.1 (0.3) ^c^	17.3 (0.6) ^d^	22.57 (1.02) ^b^

For each parameter, the same small letter in superscript within a row indicates homogeneous groups, as established by ANOVA (*p* < 0.05), comparing CB% (0, 2.5, 5, 7.5, 10, 12.5, and 15) in mixtures or extrudates. For each CB % (0, 2.5, 5, 7.5, 10, 12.5, and 15) and parameter, the same capital letter in superscript within a column indicates homogeneous groups, as established by ANOVA (*p* < 0.05), comparing mixtures and extrudates.

**Table 7:** Dietary fiber (insoluble, soluble and total) contents in unprocessed and extruded samples of corn flours (g/100 g, dry weight) (mean ± SD).

Sample	IDF	SDF	TDF
F0	7.2 (0.6) ^dA^	1.0 (0.2) ^bA^	8.2 (0.8) ^cA^
F1	10.50 (0.15) ^cA^	1.2 (0.3) ^abA^	11.8 (0.2) ^bA^
F2	11.0 (0.6) ^bcA^	1.4 (0.2) ^abA^	12.4 (0.7) ^bA^
F3	11.8 (0.5) ^bA^	1.5 (0.3) ^abA^	13.3 (0.7) ^abA^
F4	13.5 (0.2) ^aA^	1.71 (0.05) ^aA^	14.5 (0.6) ^aA^
Sample	IDF	SDF	TDF
F0E	5.7 (0.6) ^dB^	0.90 (0.08) ^bA^	6.6 (0.7) ^cB^
F1E	7.32 (0.16) ^cB^	1.20 (0.14) ^aA^	7.4 (0.2) ^cBA^
F2E	9.5 (0.9) ^bB^	1.15 (0.02) ^aB^	9.9 (0.9) ^bB^
F3E	11.39 (1.05) ^aA^	1.17 (0.15) ^aB^	12.8 (0.9) ^aA^
F4E	12.7 (0.6) ^aA^	1.17 (0.08) ^aB^	13.9 (0.7) ^aA^

IDF: insoluble dietary fiber; SDF: soluble dietary fiber; TDF: total dietary fiber. For each parameter, the same small letter in superscript within a row indicates homogeneous groups as established by ANOVA (*p* < 0.05), comparing CB% (0, 2.5, 5, 7.5, 10, 12.5, and 15) in mixtures or extrudates. For each CB % (0, 2.5, 5, 7.5, 10, 12.5, and 15) and parameter, the same capital letter in superscript within a column indicates homogeneous groups, as established by ANOVA (*p* < 0.05), comparing mixtures and extrudates.

## Data Availability

The original contributions presented in the study are included in the article, further inquiries can be directed to the corresponding author.
